# Supplementary material for: Laboratory diagnostic, epidemiological, and clinical characteristics of human leptospirosis in Okinawa Prefecture, Japan, 2003–2020
Source: PLoS Negl Trop Dis. 2021 Dec 14;15(12):e0009993. doi: 10.1371/journal.pntd.0009993 (PMC8670671; doi:10.1371/journal.pntd.0009993)
Supplement: S1 Table — (DOCX) [file pntd.0009993.s001.docx]

**S1 Table. Estimated infection source of leptospirosis patients from September 1988 to October 2003 (N = 81).**

| Estimated infection source | Recreation or labour in rivers | Agricultural work | Recreation or labor in freshwater other than rivers | Direct or indirect contact with rodents | Unknown |
| --- | --- | --- | --- | --- | --- |
| Number of patients (%) | 35 (43.2) | 17 (21.0) | 17 (21.0) | 2 (2.5) | 10 (12.3) |
