# Supplementary material for: Laboratory diagnostic, epidemiological, and clinical characteristics of human leptospirosis in Okinawa Prefecture, Japan, 2003–2020
Source: PLoS Negl Trop Dis. 2021 Dec 14;15(12):e0009993. doi: 10.1371/journal.pntd.0009993 (PMC8670671; doi:10.1371/journal.pntd.0009993)
Supplement: S2 Table — (DOCX) [file pntd.0009993.s002.docx]

**S2 Table. Comparisons of age and sex ratio of patients by estimated infection source (N = 245).**

| Estimated infection source | Recreation or labour in rivers | Agricultural work | Recreation or labor in freshwater other than rivers | Direct or indirect contact with rodents | Unknown |
| --- | --- | --- | --- | --- | --- |
| Median age [interquartile range] | 28 [18–38] | 51.5 [39.8–65.5] | 46.5 [36–55.3] | 59 [44.5–74] | 43.5 [20–59.3] |
| Percentages of male patients (number of male : female patients) | 83.1 (147 : 30) | 90.9 (20 : 2) | 94.4 (17 : 1) | 83.3 (5 : 1) | 95.5 (21 : 1) |
