# Supplementary material for: Laboratory diagnostic, epidemiological, and clinical characteristics of human leptospirosis in Okinawa Prefecture, Japan, 2003–2020
Source: PLoS Negl Trop Dis. 2021 Dec 14;15(12):e0009993. doi: 10.1371/journal.pntd.0009993 (PMC8670671; doi:10.1371/journal.pntd.0009993)
Supplement: Attachment — Submitted filename: Response to Reviewers211022.docx [file pntd.0009993.s003.docx]

**Response to Reviewers**

**Authors’ notification to all reviewers**

During the revision process, we found that there were some mistakes in the number of samples in Table 1. We are really sorry, but we carefully checked and corrected the number of samples in the table and the text.

**Comments from Reviewer #1**

**Comment 1:** a minor typo needed be fixed; Line 375: was to were, Line 360: are to is, Line 361: canyoning, Line 384: that --> those

**Reply:** We appreciate the reviewer’s corrections for our manuscript. We carefully checked the reviewer’s corrections and revised the manuscript. We also had our revised manuscript checked by professional proofreading.

**Comments from Reviewer #2**

**Comment 1:** At the end of the introduction it is required to specify the month of the end of the period.

**Reply:** According to the reviewer’s comment, we specified the month of the end of the study period (December 2020) in the Introduction section of our revised manuscript (P8L166). We also specified the month of the end of the study period in the Abstraction section (P3L52).

**Comment 2:** In Table 3. Epidemiological information on cases with confirmed leptospirosis

The column headings are offset

**Reply:** We modified the format of the Table 3 (=Table 2 in the revised manuscript).

**Comment 3:** S1 Table. Comparisons of age and sex ratio of patients by estimated infection source.

Percentages of estimated infection source of missing male patients

**Reply:** We are unsure what modifications are being suggested here. All male patients were classified into the five categories of the estimated infection source. We added the number of male and female patients in the revised S1 Table (=S2 Table in the revised manuscript).

**Comment 4:** The month of the end of the study period remains to be specified.

**Reply:** As our reply to the reviewer’s comment 1, we specified the month of the end of the study period (December 2020) in the Abstract and Introduction sections in our revised manuscript (P3L52 and P8L166).

**Comments from Reviewer #3**

**Comment 1:** The study by Kakita and collaborators describe a case series of Leptospirosis in Okinawa. It is a retrospective study that describes the epidemiology of human leptospirosis in in that region during 2003-2020. This case series is an interesting compilation of clinically diagnosed Leptospirosis patients, their diagnostic laboratory results, and their epidemiological risk factors.

Although the data is present in the manuscript, the analysis would benefit from a better description of the study population, surveillance and case definitions including inclusion/exclusion criteria, and a flow chart describing the results on the tests performed (partially described in figure 1). The authors include a STROBE checklist, but this study is a case series, therefore the criteria for a cross sectional study are not applicable. I recommend rewriting the abstract accordingly, and reorganising the methods and results sections to resemble more appropriately a case series (as reference case report studies, please see Ko et al 1999 Lancet, Ciceron et al 2000 Eur J Epidemiol, Galan et al 2021 PLoS One).

**Reply:** According to the reviewer’s comment, we modified the first subsection in the Methods section in our revised manuscript: the heading “sample collection” was changed to “Surveillance, case definition, and data collection” and modified/added the following sentences for the better descriptions of the study population, surveillance and case definitions including inclusion/exclusion criteria.

P8L169. **Surveillance, case definition, and data collection:** This retrospective study involved 531 patients who were clinically diagnosed with leptospirosis by physicians at clinics and hospitals in Okinawa Prefecture. According to the National Epidemiological Surveillance of Infectious Diseases under the Infectious Diseases Control Law of Japan, clinical samples and leptospirosis surveillance data were collected, and the clinical samples were sent to the Okinawa Prefectural Institute of Health and Environment through regional public health centers for laboratory confirmation. The inclusion criterion was clinically suspected leptospirosis cases from which at least one of the clinical samples was collected, while the exclusion criterion was the cases from which no clinical samples were collected. Since at least one clinical sample was taken from all 531 suspected cases, there were no excluded cases for laboratory diagnosis. The case definition of confirmed leptospirosis is a person with clinical symptoms compatible with leptospirosis and with laboratory confirmation of acute infection (see “Laboratory diagnostics for leptospirosis”). One of the confirmed cases was imported; the patient had contracted leptospirosis in Thailand, and the case was removed from epidemiological and clinical analyses. Seven cases were not included in the sensitivity comparison of diagnostic methods because of a lack of information.

We also modified the Figure 1: we added the number of samples in all ranks and the results of laboratory diagnosis in the lowest boxes.

According to the reviewer’s comment, we modified the Abstraction section as follows:

(Previous) **Methodology/Principal Findings:** We conducted laboratory diagnosis of 531 patients clinically suspected of leptospirosis between November 2003 and 2020. The sensitivities of culture, nested polymerase chain reaction (PCR), and microscopic agglutination test (MAT) by sampling date and clinical samples were compared. Statistical analyses were performed to compare the epidemiological and clinical features of confirmed cases. Serogroups and the species of *Leptospira* isolates were determined by *flaB* sequencing and MAT using 13 antisera. Among the 531 patients, 246 (46.3%) were laboratory-confirmed to have leptospirosis. The sensitivities of culture and PCR exceeded 65% during the first 6 days. Approximately half of the isolates were *L. interrogans* serogroup Hebdomadis. Among the confirmed cases, age 20–29 years (22.4%) and male sex (85.7%) were the most common. The most common estimated sources of infection were recreation (44.5%) and labor (27.8%) in rivers. The main clinical symptoms were fever (97.1%), myalgia (56.3%), and conjunctival hyperemia (52.2%). Headache occurred significantly more often in patients with Hebdomadis serogroup infections.

(Revised) **Methodology/Principal Findings:** We conducted a retrospective study of clinically suspected leptospirosis patients whose sample and information were sent to the Okinawa Prefectural Institute of Health and Environment from November 2003 to December 2020. Laboratory diagnosis was established using culture, nested polymerase chain reaction (PCR), and/or microscopic agglutination test (MAT) with blood, cerebrospinal fluid, and/or urine samples. Statistical analyses were performed to compare the epidemiological information, clinical features, and sensitivities of diagnostic methods among laboratory-confirmed cases. Serogroups and the species of *Leptospira* isolates were determined by *flaB* sequencing and MAT using 13 antisera.

A total of 531 clinically suspected patients were recruited, among whom 246 (46.3%) were laboratory confirmed to have leptospirosis. Among the confirmed cases, patients aged 20–29 years (22.4%) and male patients (85.7%) were the most common. The most common estimated sources of infection were recreation (44.5%) and labor (27.8%) in rivers. Approximately half of the isolates were of the *L. interrogans* serogroup Hebdomadis. The main clinical symptoms were fever (97.1%), myalgia (56.3%), and conjunctival hyperemia (52.2%). Headache occurred significantly more often in patients with Hebdomadis serogroup infections than those with other serogroup infections. The sensitivities of culture and PCR exceeded 65% during the first 6 days, while the sensitivity of MAT surpassed that of culture and PCR in the second week after onset. PCR using blood samples was a preferable method for the early diagnosis of leptospirosis.

We also reorganized the Methods and Results sections as the reviewer suggested. We stated the study population, surveillance and inclusion/exclusion criteria in the first subsection (Surveillance, case definition, and data collection) of the Methods section as we described above. In the Results section, we first outlined the results of laboratory diagnostic methods, and then described epidemiological features of confirmed cases. Since we compared clinical features of patients infected with serogroup Hebdomadis and other serogroups, we stated the results of serogroup identification before clinical features of patients.

| Previous manuscript | Revised manuscript |
| --- | --- |
| Methods | |
| Sample collection | Surveillance, case definition, and data collection |
| Laboratory diagnostics for leptospirosis | Laboratory diagnostics for leptospirosis |
| Species and serogroup identification for *Leptospira* isolates | Species and serogroup identification for *Leptospira* isolates |
| Data collection and statistical analysis | Statistical analysis |
| Ethics statements | Ethics statements |
| Results | |
| Laboratory diagnosis of leptospirosis | Summary of the results of laboratory diagnosis |
| Characterization of *Leptospira* isolates and infecting *Leptospira* serogroups deduced by MAT | Epidemiological features of the confirmed leptospirosis cases |
| Epidemiological features of the confirmed leptospirosis cases | Characterization of *Leptospira* isolates and infecting *Leptospira* serogroups deduced by MAT |
| Clinical features of the confirmed leptospirosis patients | Clinical features of the confirmed leptospirosis patients |
|  | Comparison of sensitivity of laboratory diagnostic methods |

**Comment 2:** The analysis is appropriate for the retrospective case report design. Figure one can include more detail about the tests performed and excluded cases.

**Reply:** As we described above, we added the number of samples in all ranks and the results of laboratory diagnosis in the lowest boxes in the Figure 1. Although one imported case was removed from epidemiological and clinical analyses (P8L182), all cases were subjected to laboratory diagnosis (no excluded case in the Figure 1).

**Response to Editorial Office**

**Comment:** We noticed that you used "unpublished data" in the manuscript. We do not allow these references, as the PLOS data access policy requires that all data be either published with the manuscript or made available in a publicly accessible database. Please either remove these references, or amend the supplementary material to include the referenced data.

**Reply:** According to the comment, we prepared a supplementary table (S1 Table) for the description of the estimated infection source of leptospirosis patients from September 1988 to October 2003.
